# Regulation of E3 ubiquitin ligases by homotypic and heterotypic assembly

**DOI:** 10.12688/f1000research.21253.1

**Published:** 2020-02-06

**Authors:** Vishnu Balaji, Thorsten Hoppe

**Affiliations:** 1 Institute for Genetics and Cologne Excellence Cluster on Cellular Stress Responses in Aging-Associated Diseases (CECAD), University of Cologne, Cologne, Germany; 2 Center for Molecular Medicine Cologne (CMMC), University of Cologne, Cologne, Germany

**Keywords:** C. elegans, ubiquitin, chaperone, proteostasis, E3 ligase, CHIP, RING, HECT

## Abstract

Protein ubiquitylation is essential for the maintenance of cellular homeostasis. E3 ubiquitin ligases are key components of the enzymatic machinery catalyzing the attachment of ubiquitin to substrate proteins. Consequently, enzymatic dysfunction has been associated with medical conditions including cancer, diabetes, and cardiovascular and neurodegenerative disorders. To safeguard substrate selection and ubiquitylation, the activity of E3 ligases is tightly regulated by post-translational modifications including phosphorylation, sumoylation, and ubiquitylation, as well as binding of alternative adaptor molecules and cofactors. Recent structural studies identified homotypic and heterotypic interactions between E3 ligases, adding another layer of control for rapid adaptation to changing environmental and physiological conditions. Here, we discuss the regulation of E3 ligase activity by combinatorial oligomerization and summarize examples of associated ubiquitylation pathways and mechanisms.

## Introduction

The covalent attachment of ubiquitin to substrate proteins is essential for the maintenance of organismal homeostasis by regulating diverse cellular signaling processes and protein quality control ^1^. Substrate ubiquitylation is usually mediated by the sequential activity of ubiquitin-activating enzymes (E1), ubiquitin-conjugating enzymes (E2), and ubiquitin ligases (E3). The E3 ubiquitin ligases form the largest group with more than 600 members in humans, which provide a central role in catalyzing ubiquitin conjugation to internal lysine residues of specific substrates and thereby defining their fates ^2^. Depending on the mechanism by which ubiquitin is transferred from the E2 enzyme to the substrate, E3 ligases are classified into Really Interesting New Gene (RING) finger domain-, Homologous to E6-associated protein C Terminus (HECT) domain-, or RING Between RING (RBR) domain-containing ubiquitin ligases ^3^. While RING E3s facilitate the direct transfer of ubiquitin from E2~ubiquitin intermediates to the target protein, HECT and RBR E3s contain an active-site cysteine that forms a thioester with ubiquitin before transferring it to the substrate ^3– 5^. Despite a plethora of structurally unrelated proteins, their ubiquitylation is highly selective owing to the high number and the distinctive nature of E3 ligases. Usually, one E3 ligase can target and regulate several substrate proteins ^6^. Therefore, the expression, activity, and turnover of E3 ligases is tightly regulated to prevent cellular dysfunctions ^6, 7^. E3 expression undergoes spatiotemporal control regulated by tissue-specific gene expression, gene imprinting, the cellular microenvironment, and levels of substrate protein ^8– 11^. Moreover, the activity and abundance of E3 ligases are defined by both post-translational modifications and binding of cofactors and/or adaptor molecules ^12– 14^. Besides these well-known control mechanisms, recent structural work identified an additional layer of regulation provided by homotypic and heterotypic combination of E3 ligases into oligomeric ubiquitylation complexes ^4, 5, 15, 16^. However, despite recent reports describing oligomer formation of E3 ligases, the underlying regulatory mechanisms and the physiological relevance largely remain unclear. Here we provide an overview on homotypic and heterotypic assembly of E3 ubiquitin ligases and potential implications in drug discovery and therapeutic interventions ^17^.

## Oligomer formation: a shared principle of E3 ubiquitin ligases

**Figure 1.**
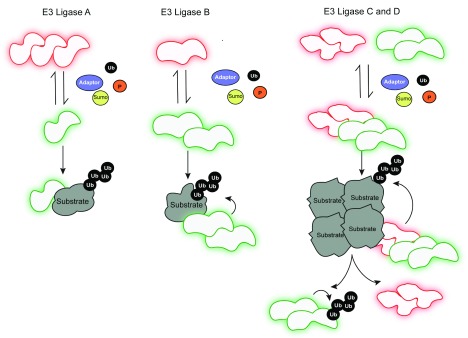
Different types of E3 ligase regulation and assembly. E3 ligase A is inactive (red) as an oligomer and converted into an active monomer (green) upon post-translational modification or binding to adaptor molecules, indicated with orange, yellow, black, and violet circles, representing phosphate (P), sumo, ubiquitin (Ub), and adaptor molecules, respectively. Conversely, E3 ligase B is inactive as a monomer and activated upon dimerization. Heterotypic interaction of inactive E3 ligase C and active E3 ligase D results in the formation of a multimeric E3 ligase complex, which is able to target oligomeric substrates for ubiquitylation. Upon substrate degradation, the remaining, active ligase D undergoes auto-ubiquitylation and turnover. The different substrates are indicated in other shapes.

Oligomer formation specifically modulates the catalytic activity of RING finger and HECT type E3 ubiquitin ligases ( Figure 1 and Table 1) ^5, 13, 15^. The HECT ligases SMURF1, NEDD4.1, and HUWE1 are negatively regulated by oligomerization, which limits the accessibility of the catalytic cysteine residues for ubiquitin binding ^5, 18, 19^. Conversely, oligomerization can also promote the catalytic function, which was shown for the HECT ligase E6AP and the RING ligases BIRC7, cIAP, TRAF6, RNF4, and Mdm2–Mdmx ^4, 5, 8, 16, 20, 21^. E3 ubiquitin ligases form different types of oligomers including homotypic interactions where one monomer binds to one or more of its respective counterparts either symmetrically, as observed for SMURF1 and E6AP ^18, 22^, or asymmetrically, as reported for the RING/U-box ligases Rad18 and CHIP ^23, 24^. In contrast, heterotypic oligomers are formed between different E3s, such as the RING ligases Brca1–Bard1 and Mdm2–Mdmx ( Figure 1) ^25, 26^. Likewise, the multi-subunit Cullin–RING E3 ubiquitin ligases (CRLs) form complex oligomeric assemblies for nuanced regulation of their activity and effective substrate recruitment ^27– 30^.

**Table 1.** List of E3 ubiquitin ligases forming oligomers.

S No	E3 ligase	Class	Oligomeric state	References
1	SMURF1	HECT	Inactive	Wan *et al*. ^36^
2	NEDD4.1	HECT	Inactive	Attali *et al*. ^31^
3	HUWE1	HECT	Inactive	Sander *et al*. ^19^
4	E6AP	HECT	Active	Ronchi *et al*. ^22^
5	BIRC7	RING	Active	Dou *et al*. ^21^
6	cIAP1	RING	Active	Mace *et al*. ^39^
7	TRAF6	RING	Active	Yin *et al*. ^40^
8	RNF4	RING	Active	Plechanovová *et al*. ^46^; Rojas-Fernandez *et al*. ^8^
9	MDM2	RING	Active	Poyurovsky *et al*. ^44^; Cheng *et al.* ^12^
10	RAD18	RING	Active	Huang *et al*. ^23^
11	Brca1	RING	Active	Brzovic *et al.* ^25^
12	Cbl-b	RING	Active	Peschard *et al.* ^35^
13	IDOL	RING	Active	Zhang *et al*. ^42^
14	SIAH1	RING	Active	Hu G and Fearon ER ^47^
15	CHIP	U-box	Active	Zhang *et al*. ^24^

Oligomer formation of E3 ligases is mechanistically regulated by post-translational modifications including phosphorylation, sumoylation, and even ubiquitylation ( Figure 1) ^8, 14, 31, 32^. For example, the HECT ligase E6AP is active in its trimeric form whereas monomerization inhibits its catalytic function, which is triggered by c-Abl kinase-dependent phosphorylation ^22, 32^. This phenomenon is intriguingly different from other HECT ligases, which are inactive as oligomers. Ronchi *et al*. reported that most HECT ligases contain a conserved α-helix, which inhibits oligomerization but is absent in E6AP immediately N-terminal to Asn ^497^. Adding evidence to this structural condition, increasing concentrations of the α-helix-related peptide abrogate the oligomerization and catalytic activity of E6AP ^22^. However, the HECT domain of E6AP is also observed to be a monomer in solution ^33^. Therefore, further studies are required to shed light on the role of monomers and oligomers as well as the stimuli for their molecular switch. Alternatively, the yeast and human HECT ligases Rsp5 and NEDD4 adopt auto-inhibitory homo-trimer conformations upon ubiquitylation ^31^. Trimerization is achieved by exposure of a hidden oligomeric interface due to the attraction of the conjugated ubiquitin to a ubiquitin-binding patch at the other side of the HECT domain. This allosteric mechanism restricts an essential motion between the N-terminal and the C-terminal lobes of the HECT domain ^34^. Similarly, dimer-dependent activation of the RING ligase Cbl-b is mediated by ubiquitin binding ^35^. The RING domain-containing SUMO-targeted ubiquitin ligase (STUbL) RNF4 is predominantly monomeric and inactive under normal conditions. Upon proteotoxic stress, poly-SUMO chains accumulate and recruit RNF4, which facilitates its dimerization and activity ^8^.

Besides post-translational modifications, homotypic and heterotypic interaction between E3 ligases is supported by adaptor proteins and specialized cofactors ( Figure 1). For instance, homodimerization of SMURF1 mediates auto-inhibition, which is disrupted upon allosteric interaction with CDH1 and CKIP ^36^. Another E3 ligase, HUWE1, has a distinct oligomerization mechanism where its active and inactive states are promoted by intramolecular and intermolecular interactions ^19^. One monomer of HUWE1 is auto-inhibited upon dimerization, which might trigger overall inhibition of its catalytic function ^19^. Interestingly, HUWE1 usually counteracts its auto-inhibitory state by an intramolecular interaction with a segment located 50 residues upstream of the dimer-binding region to remain active. HUWE1 inhibitors like p14ARF have been reported to bind to this segment and promote the auto-inhibitory dimeric conformation ^5, 19^. In contrast, the dimerization interface of cIAP1 stays in a closed inactive conformation until it is bound and stabilized by IAP antagonists such as SMAC mimetics, which open up the interface and facilitate dimerization-dependent cIAP1 activation ^4, 37– 39^. Adaptor proteins can also fine-tune the balance between dimer and oligomer assemblies of E3 ligases, as seen in CRL3. Here, the adaptor protein SPOP, which is a positive regulator of oligomerization, teams up with the negative regulator SPOPL in controlling the catalytic activity of the E3 ligase ^27^.

## Regulatory mechanisms and physiological relevance of E3 ligase assembly

The dimerization of the E3 ligase TRAF6 occurs via its RING domain, which primes for oligomerization via the coiled-coil (CC) region. This elegant assembly supports binding of the RING domains to numerous E2~ubiquitin molecules and formation of extended poly-ubiquitin chains. In addition, the CC domain of TRAF6 fosters recruitment and on-site recharging of E2 with ubiquitin without complete dissociation from the E3 ligase. This effective mechanism further increases the rate of polyubiquitin chain formation ^16, 40^. Binding of E2~ubiquitin by RING domains is also required for the backside binding of E2s like the UBCH5 family, which provide a specialized role in polyubiquitylation of substrate proteins ^15, 41^.

Regarding the homodimeric RING E3s BIRC7, RNF4, cIAP, and IDOL, both the monomer subunits are intrinsically capable of interacting with E2 enzymes ^4, 15, 42^. Whereas for the heterodimeric RING ligases BRCA1–BARD1 and RING1B–Bmi1, only one of the monomer subunits is able to interact with the E2 enzyme; the other one is mostly inactive while serving to stabilize the complex, target substrates, and support the enzymatic activity ^4^. Remarkably, Mdm2 and Mdmx assemble both Mdm2–Mdm2 and Mdmx–Mdmx homodimers *in vitro,* but when mixed together they prefer to form Mdm2–Mdmx heterodimers ^4, 43, 44^. The Mdm2–Mdmx heterodimer has the potential to form tetramers, especially to target the putative substrate p53, which is primarily a tetramer ^4, 45^. For the E3 ligases MDM2 and SIAH1, homo-oligomerization might also provide a role in auto-degradation ^14^. Remarkably, upon degradation of their substrates, the increased cellular level of these ligases triggers homo-dimerization and subsequently pushes the equilibrium towards auto-ubiquitylation *in trans* and subsequent proteasomal degradation ( Figure 1) ^14, 47, 48^. This mechanism removes the excessive E3 ligase molecules and thereby regulates the level of the enzyme. Especially in the case of Mdm2, the stringent control of E3 ligase level seems to be important to prevent tumorigenesis ^49– 51^.

As described before, the RING domain has a direct role in binding E2~ubiquitin conjugates. Interestingly, dimers of the RING ligases RNF4, cIAP, and BIRC7 have higher affinity to E2~ubiquitin than their monomeric counterparts ^15^. RING dimers preferentially bind charged E2~ubiquitin rather than E2 alone ^21^. Most monomeric RING E3 ligases possess a conserved tryptophan residue, which is critical for binding to E2~ubiquitin conjugates and optimal ligase activity, while the dimeric RING E3s present different residues at this position. Strikingly, RING dimers, when endowed with this tryptophan residue, are hyperactive ^52^. During the course of evolution, this particular tryptophan residue seemed to be modified in dimeric E3 ligases to prevent aberrant functioning and to enable regulation of the catalytic activity only by oligomer formation ^52^. It has been demonstrated that RNF4 is present in a basal inactive monomer form and only proteotoxic and genotoxic stress conditions increase polySUMO chain levels to potentially induce dimer formation and enzymatic activation ^8, 52^. As a common feature, dimeric ligases are critical for several signaling pathways and their misregulation results in cellular defects and cancer progression ^6, 50, 53, 54^.

Interestingly, the conserved U-box domain protein Ufd2p/UFD-2 functions as both an E3 and an E4 ligase ^55^. Unlike the U-box containing E3 ligase CHIP, which forms an asymmetric homodimer, UFD-2 exists as a monomer ^24, 56^. The structure of UFD-2 shows that it can readily bind to E2~ubiquitin conjugates as a monomer in a similar fashion to dimeric CHIP ^55, 56^. The question of why some proteins exist as monomers while some are dimers is addressing an interesting aspect considering that UFD-2 teams up with CHIP to enhance polyubiquitylation of the myosin assembly chaperone UNC-45 in *Caenorhabditis elegans*. Therefore, it is interesting to speculate that UFD-2 and CHIP form a heterodimeric complex providing altered substrate specificity and processing ^56^.

## Conclusion

E3 ubiquitin ligases regulate a myriad of proteins and therefore their expression and activity need to be tightly controlled to prevent dysfunction and toxicity ^6^. Besides multiple regulatory principles, oligomerization appears to be a key mechanism in the adaptation of E3 ligase activity to cellular requirements ^37, 46, 47, 55^. Ubiquitylation results in either proteolytic or non-proteolytic fates of conjugated substrates ^14^. Therefore, it is intriguing to speculate that oligomeric E3 ligases promote polyubiquitylation and proteasomal degradation, whereas oligomer disassembly supports monoubiquitylation and non-proteolytic substrate regulation ^27^. Depending on the concentration, Mdm2 is able to polyubiquitylate or monoubiquitylate p53, which results in proteasomal degradation or nuclear export ^57^. Conclusively, oligomer formation provides an elegant mechanism, which defines E3 ligase function.

Studying the underlying regulation involves various challenges. For many E3 ligases, the regulation and physiological relevance of oligomer formation is not completely understood because of the limitation of methods to follow the dynamic (dis)assembly of E3 ligase complexes *in vivo*. Indeed, many studies were performed under non-physiological conditions by analyzing the structure of recombinantly expressed protein domains *in vitro* or transgenic overexpression *in cellulo*, where the dynamic regulation is different compared to endogenous conditions. For example, studies on the yeast Rad18 RING domain or *Zebrafish* CHIP U-box domain suggested a symmetric homo-dimer assembly and altered E2 binding in contrast to results obtained for both full-length proteins ^15, 23, 58^. Another limitation is that some E3 ligases exist in a monomer–dimer transition state in solution ^15^, suggesting that binding of E2, adaptor molecules, or chaperones is able to modulate the equilibrium ^18, 59^. In addition, the expression of E3 ligases is often tissue-specifically regulated and can trigger concentration-dependent changes in oligomer formation ^5^.

The regulatory role of homotypic and heterotypic combination of E3 ligases appears to be an attractive mechanism to target for drug discovery. Indeed, IAP antagonists are known to promote cIAP dimerization and activity for treating cancer ^15, 38^. Similarly, homo- or hetero-Proteolysis-Targeting Chimeras (PROTACs) are synthetic small molecules that promote dimerization of specific E3 ligases. For example, the homobifunctional compounds CML1 and 15a induce effective dimerization of the CRL2 subunits VHL and CRBN, respectively, which results in the self-degradation of VHL and CRBN ^17, 60^. Alternatively, the heterobifunctional compounds 14a and CRBN-6-4-5-5-VHL, synthesized to target both VHL and CRBN, preferentially degrade CRBN over VHL ^61, 62^. In the case of the E3 ligase CHIP, specific peptides were shown to inhibit its dimerization and E3 ligase activity ^63^. More studies and technological advances will provide better insights and understanding of the oligomerization mechanism, which will help to design compounds to manipulate E3 ligase assembly for therapeutic applications.

## Abbreviations

CC, coiled-coil; CRL, Cullin–RING E3 ubiquitin ligase; HECT, Homologous to E6-associated protein C Terminus; RBR, RING Between RING; RING, Really Interesting New Gene.

## References

[1] Komander D Rape M: The ubiquitin code. *Annu Rev Biochem.* 2012;81:203–29. 10.1146/annurev-biochem-060310-170328 22524316

[2] Hershko A Ciechanover A Varshavsky A: Basic Medical Research Award. The ubiquitin system. *Nat Med.* 2000;6(10):1073–81. 10.1038/80384 11017125

[3] Zheng N Shabek N: Ubiquitin Ligases: Structure, Function, and Regulation. *Annu Rev Biochem.* 2017;86:129–57. 10.1146/annurev-biochem-060815-014922 28375744

[4] Metzger MB Pruneda JN Klevit RE: RING-type E3 ligases: master manipulators of E2 ubiquitin-conjugating enzymes and ubiquitination. *Biochim Biophys Acta.* 2014;1843(1):47–60. 10.1016/j.bbamcr.2013.05.026 23747565 PMC4109693

[5] Sluimer J Distel B: Regulating the human HECT E3 ligases. *Cell Mol Life Sci.* 2018;75(17):3121–41. 10.1007/s00018-018-2848-2 29858610 PMC6063350

[6] Balaji V Pokrzywa W Hoppe T: Ubiquitylation Pathways In Insulin Signaling and Organismal Homeostasis. *Bioessays.* 2018;40(5):e1700223. 10.1002/bies.201700223 29611634

[7] Kevei É Pokrzywa W Hoppe T: Repair or destruction-an intimate liaison between ubiquitin ligases and molecular chaperones in proteostasis. *FEBS Lett.* 2017;591(17):2616–35. 10.1002/1873-3468.12750 28699655 PMC5601288

[8] Rojas-Fernandez A Plechanovová A Hattersley N: SUMO chain-induced dimerization activates RNF4. *Mol Cell.* 2014;53(6):880–92. 10.1016/j.molcel.2014.02.031 24656128 PMC3991395

[9] Song R Peng W Zhang Y: Central role of E3 ubiquitin ligase MG53 in insulin resistance and metabolic disorders. *Nature.* 2013;494(7437):375–9. 10.1038/nature11834 23354051

[10] Nagarajan A Petersen MC Nasiri AR: MARCH1 regulates insulin sensitivity by controlling cell surface insulin receptor levels. *Nat Commun.* 2016;7:12639. 10.1038/ncomms12639 27577745 PMC5013666

[11] Mabb AM Judson MC Zylka MJ: Angelman syndrome: insights into genomic imprinting and neurodevelopmental phenotypes. *Trends Neurosci.* 2011;34(6):293–303. 10.1016/j.tins.2011.04.001 21592595 PMC3116240

[12] Cheng Q Cross B Li B: Regulation of MDM2 E3 ligase activity by phosphorylation after DNA damage. *Mol Cell Biol.* 2011;31(24):4951–63. 10.1128/MCB.05553-11 21986495 PMC3233024

[13] Deshaies RJ Joazeiro CA: RING domain E3 ubiquitin ligases. *Annu Rev Biochem.* 2009;78:399–434. 10.1146/annurev.biochem.78.101807.093809 19489725

[14] de Bie P Ciechanover A: Ubiquitination of E3 ligases: self-regulation of the ubiquitin system via proteolytic and non-proteolytic mechanisms. *Cell Death Differ.* 2011;18(9):1393–402. 10.1038/cdd.2011.16 21372847 PMC3178436

[15] Budhidarmo R Nakatani Y Day CL: RINGs hold the key to ubiquitin transfer. *Trends Biochem Sci.* 2012;37(2):58–65. 10.1016/j.tibs.2011.11.001 22154517

[16] Hu L Xu J Xie X: Oligomerization-primed coiled-coil domain interaction with Ubc13 confers processivity to TRAF6 ubiquitin ligase activity. *Nat Commun.* 2017;8(1):814. 10.1038/s41467-017-01290-0 28993672 PMC5634496

[17] Ottis P Toure M Cromm PM: Assessing Different E3 Ligases for Small Molecule Induced Protein Ubiquitination and Degradation. *ACS Chem Biol.* 2017;12(10):2570–8. 10.1021/acschembio.7b00485 28767222

[18] Xie P Zhang M He S: The covalent modifier Nedd8 is critical for the activation of Smurf1 ubiquitin ligase in tumorigenesis. *Nat Commun.* 2014;5:3733. 10.1038/ncomms4733 24821572

[19] Sander B Xu W Eilers M: A conformational switch regulates the ubiquitin ligase HUWE1. *elife.* 2017;6:pii: e21036. 10.7554/eLife.21036 28193319 PMC5308896

[20] Ries LK Sander B Deol KK: Analysis of ubiquitin recognition by the HECT ligase E6AP provides insight into its linkage specificity. *J Biol Chem.* 2019;294(15):6113–29. 10.1074/jbc.RA118.007014 30737286 PMC6463701

[21] Dou H Buetow L Sibbet GJ: BIRC7-E2 ubiquitin conjugate structure reveals the mechanism of ubiquitin transfer by a RING dimer. *Nat Struct Mol Biol.* 2012;19(9):876–83. 10.1038/nsmb.2379 22902369 PMC3880866

[22] Ronchi VP Klein JM Edwards DJ: The active form of E6-associated protein (E6AP)/UBE3A ubiquitin ligase is an oligomer. *J Biol Chem.* 2014;289(2):1033–48. 10.1074/jbc.M113.517805 24273172 PMC3887172

[23] Huang A Hibbert RG de Jong RN: Symmetry and asymmetry of the RING-RING dimer of Rad18. *J Mol Biol.* 2011;410(3):424–35. 10.1016/j.jmb.2011.04.051 21549715

[24] Zhang M Windheim M Roe SM: Chaperoned ubiquitylation--crystal structures of the CHIP U box E3 ubiquitin ligase and a CHIP-Ubc13-Uev1a complex. *Mol Cell.* 2005;20(4):525–38. 10.1016/j.molcel.2005.09.023 16307917

[25] Brzovic PS Rajagopal P Hoyt DW: Structure of a BRCA1-BARD1 heterodimeric RING-RING complex. *Nat Struct Biol.* 2001;8(10):833–7. 10.1038/nsb1001-833 11573085

[26] Cheng Q Chen L Li Z: ATM activates p53 by regulating MDM2 oligomerization and E3 processivity. *EMBO J.* 2009;28(24):3857–67. 10.1038/emboj.2009.294 19816404 PMC2797053

[27] Errington WJ Khan MQ Bueler SA: Adaptor protein self-assembly drives the control of a cullin-RING ubiquitin ligase. *Structure.* 2012;20(7):1141–53. 10.1016/j.str.2012.04.009 22632832

[28] Canning P Cooper CD Krojer T: Structural basis for Cul3 protein assembly with the BTB-Kelch family of E3 ubiquitin ligases. *J Biol Chem.* 2013;288(11):7803–14. 10.1074/jbc.M112.437996 23349464 PMC3597819

[29] Ahn J Novince Z Concel J: The Cullin-RING E3 ubiquitin ligase CRL4-DCAF1 complex dimerizes via a short helical region in DCAF1. *Biochemistry.* 2011;50(8):1359–67. 10.1021/bi101749s 21226479 PMC3072279

[30] Chew EH Poobalasingam T Hawkey CJ: Characterization of cullin-based E3 ubiquitin ligases in intact mammalian cells--evidence for cullin dimerization. *Cell Signal.* 2007;19(5):1071–80. 10.1016/j.cellsig.2006.12.002 17254749

[31] Attali I Tobelaim WS Persaud A: Ubiquitylation-dependent oligomerization regulates activity of Nedd4 ligases. *EMBO J.* 2016;36(4):425–40. 10.15252/embj.201694314 28069708 PMC5437815

[32] Chan AL Grossman T Zuckerman V: c-Abl phosphorylates E6AP and regulates its E3 ubiquitin ligase activity. *Biochemistry.* 2013;52(18):3119–29. 10.1021/bi301710c 23581475

[33] Huang L Kinnucan E Wang G: Structure of an E6AP-UbcH7 complex: insights into ubiquitination by the E2-E3 enzyme cascade. *Science.* 1999;286(5443):1321–6. 10.1126/science.286.5443.1321 10558980

[34] Verdecia MA Joazeiro CAP Wells NJ: Conformational flexibility underlies ubiquitin ligation mediated by the WWP1 HECT domain E3 ligase. *Mol Cell.* 2003;11(1):249–59. 10.1016/S1097-2765(02)00774-8 12535537

[35] Peschard P Kozlov G Lin T: Structural basis for ubiquitin-mediated dimerization and activation of the ubiquitin protein ligase Cbl-b. *Mol Cell.* 2007;27(3):474–85. 10.1016/j.molcel.2007.06.023 17679095

[36] Wan L Zou W Gao D: Cdh1 regulates osteoblast function through an APC/C-independent modulation of Smurf1. *Mol Cell.* 2011;44(5):721–33. 10.1016/j.molcel.2011.09.024 22152476 PMC3240853

[37] Feltham R Bettjeman B Budhidarmo R: Smac mimetics activate the E3 ligase activity of cIAP1 protein by promoting RING domain dimerization. *J Biol Chem.* 2011;286(19):17015–28. 10.1074/jbc.M111.222919 21393245 PMC3089546

[38] Dueber EC Schoeffler AJ Lingel A: Antagonists induce a conformational change in cIAP1 that promotes autoubiquitination. *Science.* 2011;334(6054):376–80. 10.1126/science.1207862 22021857

[39] Mace PD Linke K Feltham R: Structures of the cIAP2 RING domain reveal conformational changes associated with ubiquitin-conjugating enzyme (E2) recruitment. *J Biol Chem.* 2008;283(46):31633–40. 10.1074/jbc.M804753200 18784070

[40] Yin Q Lin SC Lamothe B: E2 interaction and dimerization in the crystal structure of TRAF6. *Nat Struct Mol Biol.* 2009;16(6):658–66. 10.1038/nsmb.1605 19465916 PMC2834951

[41] Sakata E Satoh T Yamamoto S: Crystal structure of UbcH5b~ubiquitin intermediate: insight into the formation of the self-assembled E2~Ub conjugates. *Structure.* 2010;18(1):138–47. 10.1016/j.str.2009.11.007 20152160

[42] Zhang L Fairall L Goult BT: The IDOL-UBE2D complex mediates sterol-dependent degradation of the LDL receptor. *Genes Dev.* 2011;25(12):1262–74. 10.1101/gad.2056211 21685362 PMC3127428

[43] Uldrijan S Pannekoek WJ Vousden KH: An essential function of the extreme C-terminus of MDM2 can be provided by MDMX. *EMBO J.* 2006;26(1):102–12. 10.1038/sj.emboj.7601469 17159902 PMC1782374

[44] Poyurovsky MV Priest C Kentsis A: The Mdm2 RING domain C-terminus is required for supramolecular assembly and ubiquitin ligase activity. *EMBO J.* 2007;26(1):90–101. 10.1038/sj.emboj.7601465 17170710 PMC1782380

[45] Friedman PN Chen X Bargonetti J: The p53 protein is an unusually shaped tetramer that binds directly to DNA. *Proc Natl Acad Sci U S A.* 1993;90(8):3319–23. 10.1073/pnas.90.8.3319 8475074 PMC46291

[46] Plechanovová A Jaffray EG McMahon SA: Mechanism of ubiquitylation by dimeric RING ligase RNF4. *Nat Struct Mol Biol.* 2011;18(9):1052–9. 10.1038/nsmb.2108 21857666 PMC3326525

[47] Hu G Fearon ER: Siah-1 N-terminal RING domain is required for proteolysis function, and C-terminal sequences regulate oligomerization and binding to target proteins. *Mol Cell Biol.* 1999;19(1):724–32. 10.1128/MCB.19.1.724 9858595 PMC83929

[48] Fang S Jensen JP Ludwig RL: Mdm2 is a RING finger-dependent ubiquitin protein ligase for itself and p53. *J Biol Chem.* 2000;275(12):8945–51. 10.1074/jbc.275.12.8945 10722742

[49] Girnita L Girnita A Larsson O: Mdm2-dependent ubiquitination and degradation of the insulin-like growth factor 1 receptor. *Proc Natl Acad Sci U S A.* 2003;100(14):8247–52. 10.1073/pnas.1431613100 12821780 PMC166214

[50] Wade M Li YC Wahl GM: MDM2, MDMX and p53 in oncogenesis and cancer therapy. *Nat Rev Cancer.* 2013;13(2):83–96. 10.1038/nrc3430 23303139 PMC4161369

[51] Karni-Schmidt O Lokshin M Prives C: The Roles of MDM2 and MDMX in Cancer. *Annu Rev Pathol.* 2016;11:617–44. 10.1146/annurev-pathol-012414-040349 27022975 PMC6028239

[52] Sarkar S Behera AP Borar P: Designing active RNF4 monomers by introducing a tryptophan: avidity towards E2∼Ub conjugates dictates the activity of ubiquitin RING E3 ligases. *Biochem J.* 2019;476(10):1465–82. 10.1042/BCJ20180883 31048496

[53] Yoo L Yoon AR Yun CO: Covalent ISG15 conjugation to CHIP promotes its ubiquitin E3 ligase activity and inhibits lung cancer cell growth in response to type I interferon. *Cell Death Dis.* 2018;9(2): 97. 10.1038/s41419-017-0138-9 29367604 PMC5833375

[54] Zhang L Liu L He X: CHIP promotes thyroid cancer proliferation via activation of the MAPK and AKT pathways. *Biochem Biophys Res Commun.* 2016;477(3):356–62. 10.1016/j.bbrc.2016.06.101 27342662

[55] Hoppe T Cassata G Barral JM: Regulation of the myosin-directed chaperone UNC-45 by a novel E3/E4-multiubiquitylation complex in *C. elegans*. *Cell.* 2004;118(3):337–49. 10.1016/j.cell.2004.07.014 15294159

[56] Nordquist KA Dimitrova YN Brzovic PS: Structural and functional characterization of the monomeric U-box domain from E4B. *Biochemistry.* 2010;49(2):347–55. 10.1021/bi901620v 20017557 PMC2806929

[57] Li M Brooks CL Wu-Baer F: Mono- versus polyubiquitination: differential control of p53 fate by Mdm2. *Science.* 2003;302(5652):1972–5. 10.1126/science.1091362 14671306

[58] Xu Z Devlin KI Ford MG: Structure and interactions of the helical and U-box domains of CHIP, the C terminus of HSP70 interacting protein. *Biochemistry.* 2006;45(15):4749–59. 10.1021/bi0601508 16605243

[59] Graf C Stankiewicz M Nikolay R: Insights into the conformational dynamics of the E3 ubiquitin ligase CHIP in complex with chaperones and E2 enzymes. *Biochemistry.* 2010;49(10):2121–9. 10.1021/bi901829f 20146531

[60] Maniaci C Hughes SJ Testa A: Homo-PROTACs: bivalent small-molecule dimerizers of the VHL E3 ubiquitin ligase to induce self-degradation. *Nat Commun.* 2017;8(1): 830. 10.1038/s41467-017-00954-1 29018234 PMC5635026

[61] Steinebach C Kehm H Lindner S: PROTAC-mediated crosstalk between E3 ligases. *Chem Commun (Camb).* 2019;55(12):1821–4. 10.1039/C8CC09541H 30672516

[62] Girardini M Maniaci C Hughes SJ: Cereblon versus VHL: Hijacking E3 ligases against each other using PROTACs. *Bioorg Med Chem.* 2019;27(12):2466–79. 10.1016/j.bmc.2019.02.048 30826187 PMC6561380

[63] Nikolay R Wiederkehr T Rist W: Dimerization of the human E3 ligase CHIP via a coiled-coil domain is essential for its activity. *J Biol Chem.* 2004;279(4):2673–8. 10.1074/jbc.M311112200 14610072

